# Remote Sensing of Urban Microclimate Change in L’Aquila City (Italy) after Post-Earthquake Depopulation in an Open Source GIS Environment

**DOI:** 10.3390/s17020404

**Published:** 2017-02-19

**Authors:** Valerio Baiocchi, Fabio Zottele, Donatella Dominici

**Affiliations:** 1 Department of Civil, Constructional and Environmental Engineeering (DICEA), Sapienza University of Rome, I-00184 Rome, Italy; 2 Fondazione Mach, Centre for Technology Transfer, I-38010 S. Michele all’Adige (TN), Italy; fabio.zottele@fmach.it; 3 Department of Civil, Construction-Architectural and Environmental Engineering (DICEAA), University of L’Aquila, I-67100 L’Aquila, Italy; donatella.dominici@univaq.it

**Keywords:** Landsat, L’Aquila, thermal correction, urban heating, open source, earthquake

## Abstract

This work reports a first attempt to use Landsat satellite imagery to identify possible urban microclimate changes in a city center after a seismic event that affected L’Aquila City (Abruzzo Region, Italy), on 6 April 2009. After the main seismic event, the collapse of part of the buildings, and the damaging of most of them, with the consequence of an almost total depopulation of the historic city center, may have caused alterations to the microclimate. This work develops an inexpensive work flow—using Landsat Enhanced Thematic Mapper Plus (ETM+) scenes—to construct the evolution of urban land use after the catastrophic main seismic event that hit L’Aquila. We hypothesized, that, possibly, before the event, the temperature was higher in the city center due to the presence of inhabitants (and thus home heating); while the opposite case occurred in the surrounding areas, where new settlements of inhabitants grew over a period of a few months. We decided not to look to independent meteorological data in order to avoid being biased in their investigations; thus, only the smallest dataset of Landsat ETM+ scenes were considered as input data in order to describe the thermal evolution of the land surface after the earthquake. We managed to use the Landsat archive images to provide thermal change indications, useful for understanding the urban changes induced by catastrophic events, setting up an easy to implement, robust, reproducible, and fast procedure.

## 1. Introduction

**Figure 1:**
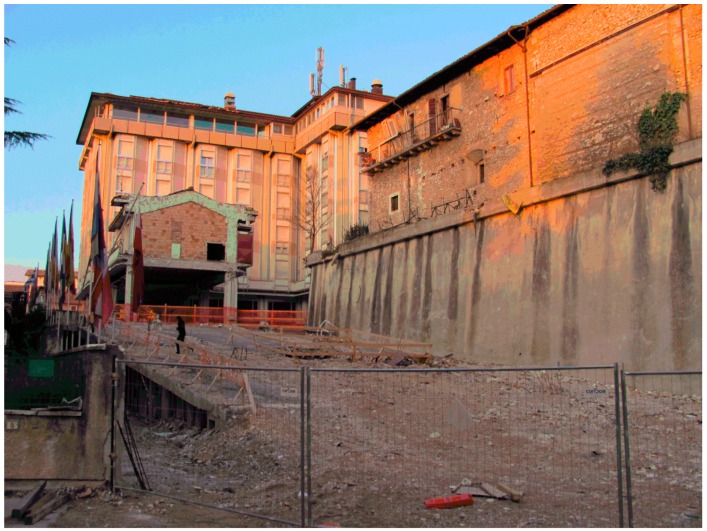
The city center after the event.

The historic center of L’Aquila is still largely uninhabited after a major seismic event on 6 April 2009 (Figure 1); thus, it may have undergone a change in its microclimate due to the sudden absence of inhabitants, which may have caused a lower impact in heating during winter. It is likely that an equal but opposite change may have occurred in areas of new settlements, originating after the seismic event.

Broad literature describes procedures used, using commercial software, to evaluate the urban heat island effect and its changes [1,2,3]; however, nowadays, free and open source software offers the possibility of developing a complete workflow to describe such dynamics [4]. The choice to use freely-available remote-sensed images, and the free and open source tools for Geographic Information System (GIS) analysis of data could aid local governance in observing the spatial dynamics of urban areas, and to determine successful policies to manage such events in the long-term, and at low costs. On the other hand, there is not an extensive literature on the use of remote sensing in order to monitor depopulation: In Reference [5], depopulation is estimated using nighttime light images; in Reference [6], instead, land-cover maps using Landsat images were used; and, finally, in Reference [7], an exposure model at a global scale, suitable for different geo-hazards, using teledetected images, was implemented.

**Figure 2:**
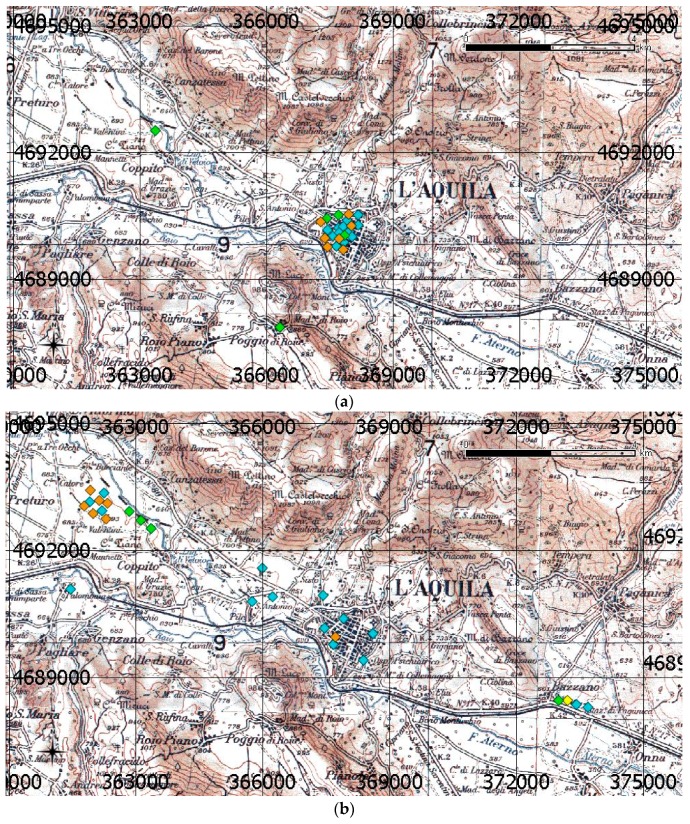
L’Aquila, before (**a**) and after (**b**) the main seismic event: Overlapped grid is Universal Transverse Mercator Projection (UTM) WGS84/ETRF00. Scale bar (upper right) is 4 km, and the north-pointing arrow (lower left) indicates the geographic north.

Satellite imagery was only used in the post-earthquake, L’Aquila City scenario, to detect damaged buildings for a quick damage assessment [8,9,10] using the geometric and thematic capabilities of Very High Resolution (VHR) images. To the best of the authors’ knowledge, no use of satellite thermal images on this area, in order to monitor depopulation, is known. Before the main shock, the inhabitants of L’Aquila numbered around 16,000, about 10,000 inhabitants, plus the university student population, which was approximately 6000 residents [11]. Some decommissioned industrial areas were present, neighboring the city, along the valley that crosses the city from NW to SE. The inhabitants of the center were relocated to areas neighboring the old city by two projects: C.A.S.E. (Project for sustainable and earthquake safe houses: 13,000 inhabitants) and M.A.P. (project for temporary housing modules: 2800 inhabitants). After the event, development of the city following the morphology of the NW to SE valley took place; thus, new residential and business buildings were relocated along the NW to SE direction, the center of the city almost empty. This trend can be observed, for example, in Figure 2a,b [11], where the public offices of national agencies (orange in Figure 2a,b), local agencies, such as prefecture and regional administration, courts (blue in Figure 2a,b), and university buildings (green in Figure 2a,b) are represented, before and after the event; the historic center is visible in the figures. A rigorous delineation of the urbanized area, after the event, is still not possible. 

It can be easily noticed that, before the seismic event, the economic and social center of the city was concentrated in a very narrow area of the historic city center (Figure 2a).

After the earthquake, most of the public offices were relocated to a surrounding area where unused industrial buildings were available (Figure 2b), leaving the center almost deserted (see Figure 1).

In this study, we set the hypothesis that the sudden relocation of houses and offices changed the human-induced micro-climate, and we used remotely-sensed images in order to track the spatio-temporal evolution of the heat island effect. 

## 2. Materials and Methods

**Figure 3:**
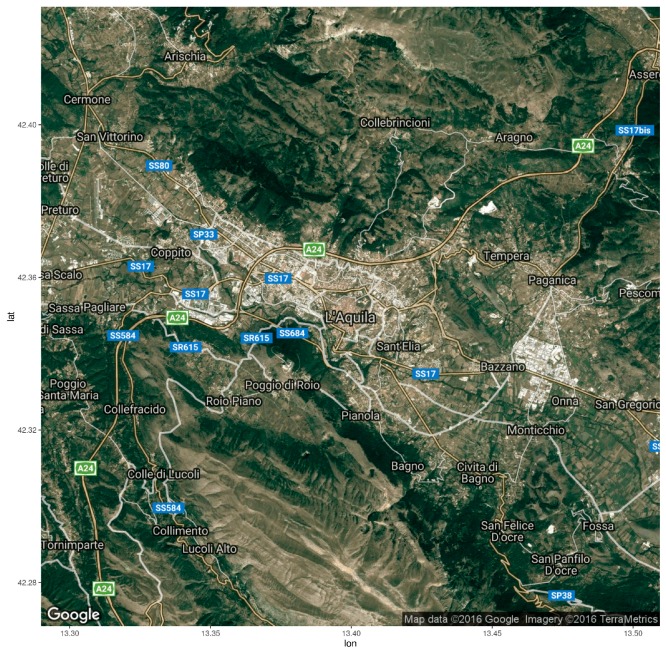
Study area of L’Aquila and its neighboring areas. Scenes copyrighted by Google Imagery, Mapdata, and Terrametrics. The image was reprojected from Spherical Mercator to WGS84; image is around 20 km in width.

The image set consists of 11 Landsat ETM+ scenes, all in the same path and row (Table 1). Each image covers the area of the central part of Italy: at the center of each image lies the urban area of Aquila (42°20′55″ N 13°23′54″ E) and the neighboring villages. The elevation of the entire area ranges between 559 and 2508 m above the sea level, with different combinations of urban and agricultural landscapes, different types of forest coverage, and bare soils (Figure 3).

The Digital Terrain Model (DTM) was obtained from the Shuttle Radar Topography Mission (SRTM) dataset, freely available online, and has a spatial resolution of 90 m [12]. 

**Figure 4:**
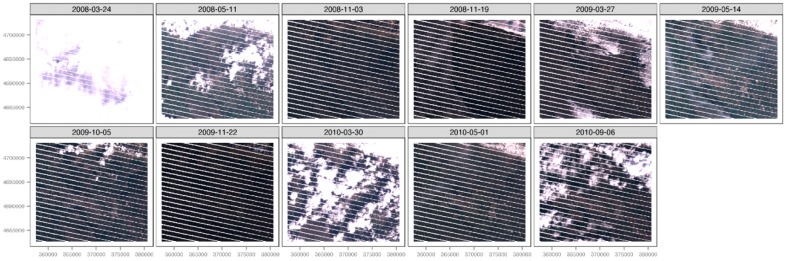
The complete image set of the Landsat ETM+ scenes where the three bands (red, green blue) acquired in the visible spectrum were blended together (Red, Blue and Green (RGB) composite), projected to UTM33N WGS84 datum, images are around 23 km in width.

Table 1 reports the attributes of the image set used in the analyses (Figure 4). The time of acquisition (Time) is expressed in Coordinated Universal Time. Latitude and Longitude refer to the center of each image and are expressed in geographic coordinates (decimal degrees) in the WGS84 datum.

It can be observed that all the images were acquired, almost at the same time, around 8:37 a.m. local time (GMT + 1), when domestic and office heating were surely being used. 

Starting from the Landsat data, a procedure to estimate the Land Surface Temperature (LST) is presented. 

Among the many free and open source tools for data analysis, we decided to choose the GRASS GIS software (GRASS stands for Geographic Resources Analysis Support System) and the R statistical software. These two tools can be easily coupled together to run geographic and statistical analyses on data in one seamless environment [13].

In order to filter the pixels where the Scan Line Corrector failed (SLC-off), the corresponding SLC-off mask was applied to each of the 9 bands of the Landsat ETM+ image.

Radiometric correction is applied to all bands of every Landsat scene, converting the Digital Numbers (DN) to at-sensor (or top-of-atmosphere) spectral radiance (L), using Equation (1). The obtained values must be corrected for solar variability, caused by annual changes in the Earth–Sun (Esun) distance (d), producing unitless, top-of-atmosphere reflectance (ρAS, Equation (2)) using data on solar intensity. Esun is the band-specific exo-atmospheric solar constant and is the solar elevation angle. 

The thermal bands are first converted from DN to ‘at-sensor radiance’, and then to effective at-sensor temperature in Kelvin. The values involved in these transformations are obtained from the metadata available for each scene [14].

(1) $$L=\left(\frac{L_{max}-L_{min}}{DN_{max}-DN_{min}}\right)\left(DN-DN_{min}\right)+L_{min},$$

(2) $$\rho_{AS}=\frac{\pi d^{2}L}{E_{sun}\cos\left(90-\theta_{s}\right)}$$

The i.landsat.toar GRASS module [15] has been used to perform the radiometric correction of the images, and was used for the calculation of the top-of-atmosphere radiance and top-of-atmosphere reflectance. In order to calculate the cloud-cover contamination, obtained from both the reflective and thermal proprieties of the scene, we used the i.landsat.acca GRASS module [16,17]. The cloud-cover signature was then used to mask each Landsat scene.

For the application developed in this work, the values of ground reflectance are needed, thus, atmospheric correction is required. We chose an absolute atmospheric method to correct the image, relying on a mechanistic understanding of atmospheric effects, deducing the values for the atmospheric parameters from the information contained within the scene itself, rather than using externally-measured data and individually adjusting each image. In brief, the conversion of the at-sensor radiance to atmospherically-corrected surface reflectance is obtained using Equation (3), where the values of *T_z_*, *T_v_*, *E_down_*, and *L_haze_* must be determined.

(3) $$\rho =\frac{\pi d^{2}\left(L-L_{haze}\right)}{T_{v}\left(E_{sun}\cos\left(90-\theta_{s}\right)T_{z}+E_{down}\right)}$$

Among the methods available, we chose the Dark Object Subtraction model (DOS), which performs a re-calibration of the image, assuming that, for pixels with low reflectance values, any apparent reflectance should be due to atmospheric scattering effects [18,19]. The DOS (Equation (4)) model searches for the lowest DN value found in a fixed number of pixels, and this selected DN, the Starting Haze Value (SHV), is converted into radiance. It is unlikely that images contain pixels that are completely black, thus, correction considers a 1% reflectance (*L*_1%_) of these areas [14].

(4) $$L_{1\%}=0.01\frac{E_{sun}\cos\left(90-\theta_{s}\right)}{\pi d^{2}}\;L_{haze}=SHV_{rad}-L_{1\%},$$

The modified Dark Object Subtraction model was applied in order to incorporate the effects of atmospheric aerosols in the atmosphere [20]. This method works iteratively, following Equation (5).

(5) $$\tau =-\cos\left(\theta_{z}\right)ln\left(1-\frac{L_{min}-0.01\frac{T_{v}}{\pi }\left(E_{down}+\frac{\cos(\theta_{z})d^{2}T_{z}}{E_{sun}}\right)}{\frac{\cos\left(\theta_{z}\right)d^{2}}{E_{sun}}}\right)\;T_{v}=e^{-\tau }\;T_{z}=e^{-\frac{\tau }{\cos\left(\theta_{z}\right)}},$$

In order to obtain the LST maps, the Land Surface Emissivity (LSE) maps were calculated using a semi-empirical method (SEM), based on the Normalized Difference Vegetation Index (NDVI, Equation (6)): Among the different approaches found in literature, such as those described in References [21,22,23], the one based on NDVI Thresholds and Vegetation Proportion (PV, Equation (7)) has been chosen [24]. A detailed description of the method can be found in References [2,25]. This method is preferred among all others because it automates the land-cover detection, and should adjust for seasonal variability in the forested landscape.

(6) $$NDVI=\frac{NIR-VIS}{NIR+VIS},$$

(7) $$P_{v}=\left(\frac{NDVI-NDVI_{min}}{NDVI_{max}-NDVI_{min}}\right),$$

Eventually, the LST maps were estimated using the procedure found in References [2,26], and using the radiative transfer equation (RTE, Equation (8)), where *B*(*T_s_*) is the black-body radiance given by Plank’s law, *T_s_* is the LST estimated for the pixel, and *ε* is the land surface emissivity.

(8) $$L=(\varepsilon B(T_{s})+\left(1-\varepsilon \right)L↓)\tau +L↑,$$

The down-welling (*L*↓) and up-welling (*L*↑) atmospheric radiance, and the transmittivity between the surface and the sensor, needed for the LST computation, were retrieved using Atmospheric Correction Parameter Calculator software, available online, which is based on a model (MODTRAN, MODerate resolution atmospheric TRANSmission) that simulates the emission and adsorption of electromagnetic radiation from 0.2 to 100 μm spectral range (from middle ultraviolet to far infrared) [27,28].

**Figure 5:**
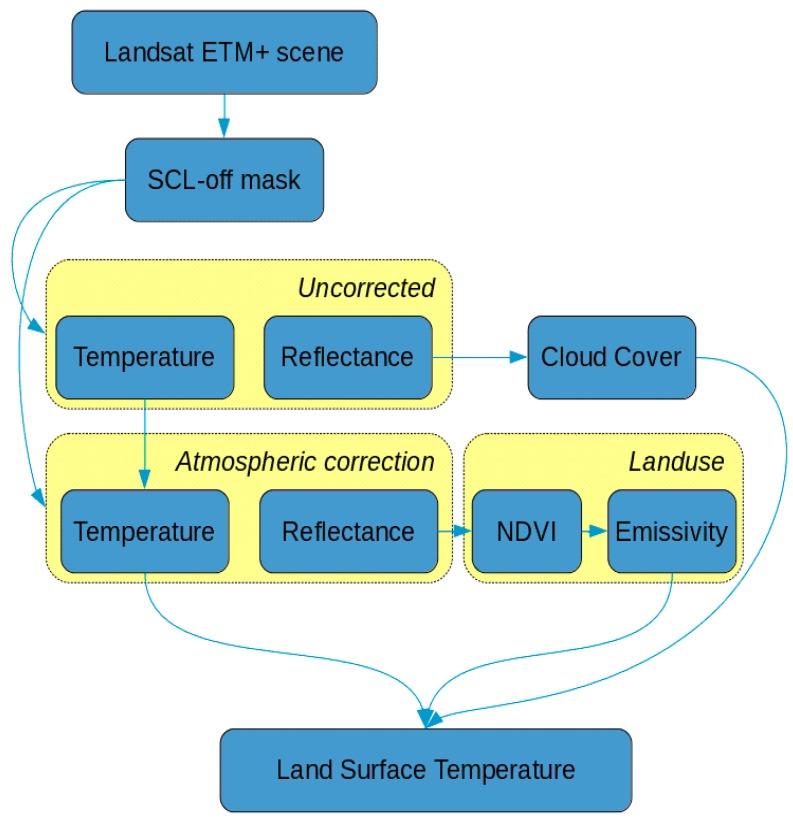
The workflow of the procedure adopted for image processing.

After processing, each LST map was cropped to the study area. The complete flowchart of the procedure is shown in Figure 5.

As we are not interested in the estimation of surface temperature, but, rather, on the description of urban exploitation, the LST of each scene was re-scaled in the range of [–1, 1] in order to enhance the visual contrast of the spatial gradient of the LST among the different land uses, and to reduce the inter-annual and intra-annual variability of LST due to meteorological conditions. 

This post-processing was performed using R software, extended with the *raster*, *ggplot2*, and *ggmap* packages [29,30,31,32].

## 3. Results

**Figure 6:**
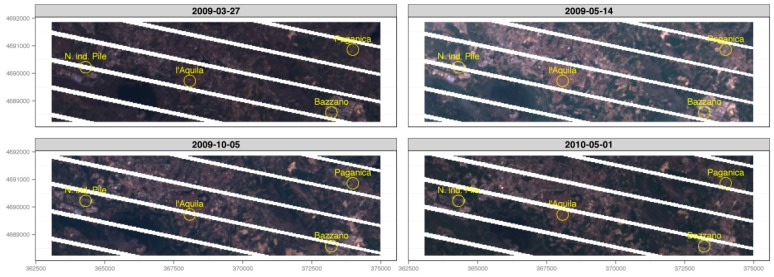
RGB composite of the four image-sets used for Land Surface Temperature estimation Scan Line corrector (SCL)-off mask applied, no cloud cover assessment); images are around 12 km in width.

**Figure 7:**
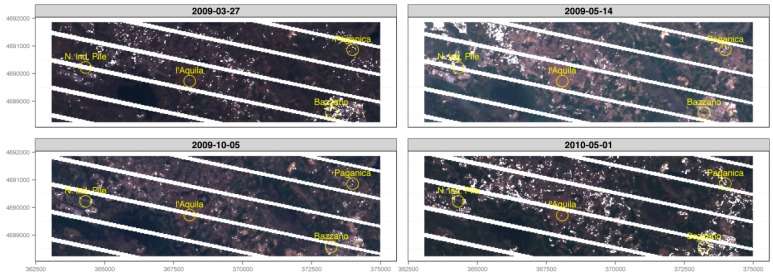
RGB composite of the four image-sets used for Land Surface Temperature estimation, with both SCL-off and cloud cover assessment masks. Images are around 12 km in width.

**Figure 8:**
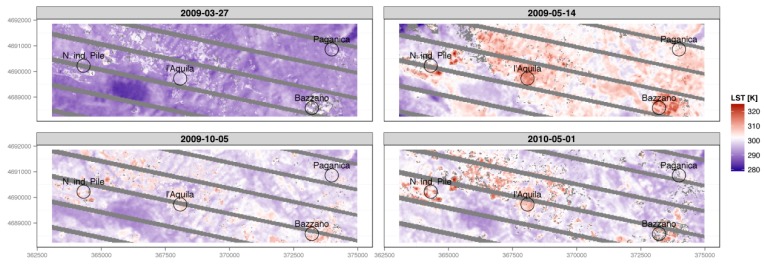
Land Surface Temperature Maps (LST) without rescale. The gray pixels correspond to the SCL-off and cloud cover assessment masks. Images are around 12 km in width.

As the combined effects of cloud cover and SLC-off greatly degraded the informative power of seven out of 11 LST maps, only four scenes (Figure 6 and Figure 7) were used to draw a qualitative analysis of the dynamics of the urban developing of Aquila and its neighbors, by using the urban micro-climate evolution as an index of the evolution (Figure 8).

**Figure 9:**
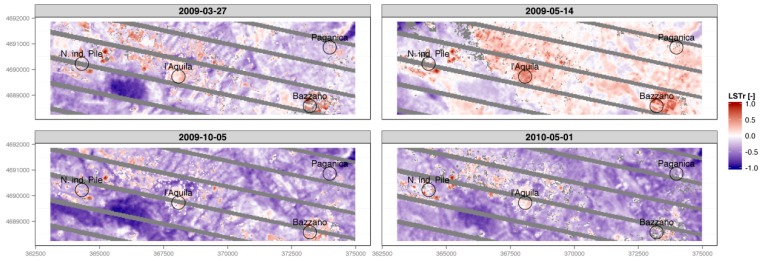
Land Surface Temperature Maps with rescale applied (LSTr). The gray pixels correspond to the SCL-off and cloud cover assessment masks. Images are around 12 km in width.

A noticeable amount of qualitative information can be drawn from the interpretation of the LST-rescaled maps (LSTr, Figure 9). Although the algorithm based on NDVI values is one of the simplest methods available for obtaining Land Surface Emissivity, it proved to be reliable in identifying the different landscapes of the study area, and the overall dynamics in urban landscape evolution after the earthquake.

The first LSTr map, which refers to the first Landsat scene available before the earthquake, is used as reference for the other three scenes, which recorded the events that followed. In this first LSTr map, urban structures (towns and roads) are effectively matched by pixels with higher temperature values. One month after the earthquake, and as of then, these structures changed remarkably: The Aquila town center is still visible, but the values of LSTr are more similar to the values of the surrounding landscapes.

Particularly interesting are the high values of LSTr recorded in the pixels near the area of Coppito, the industrial district of Bazzano, and the industrial district Pile (Figure 9). For these three areas, the values clearly remained high, and are distinct from those of surrounding pixels, before and after the disaster. For the Coppito area, this evidence could be explained by the forced displacement of survivors into existing military structures in the aftermath of the earthquake. 

The explanation for the constant high values of LSTr in the two industrial areas is linked to the more resistant structures, built using anti-seismic criteria. These earthquake-resistant building were used by the local university and other public agencies after the disaster (Figure 2b).

On the other hand, the destructive aftermath of the earthquake can be quickly identified by matching the first LSTr map with the other three, focusing on the towns of Paganica and Tempera (northeast of L’Aquila (Figure 9)): The urban structures of the two villages disappear from the LSTr time series. Indeed, it is well-known that Paganica and Tempera were severely hit by the disaster and remain uninhabited.

## 4. Discussion and Further Developments

This work aims at the development of a parsimonious work-flow for processing Landsat Enhanced Thematic Mapper Plus scenes (ETM+) in order to track changes to the urban micro-climate of L’Aquila City and neighboring towns after an earthquake event on 6 April 2009.

We know that the historic center of L’Aquila, which is still largely unpopulated, was suddenly abandoned after the event, as were some neighboring towns. However, some new towns have been built in order to help people recover, thus reshaping the urban structure of the area.

The idea underlying this work is that the forced relocation of inhabitants may have caused a lower impact of heating in winter, and of air conditioning in summer, and that these changes could potentially be reflected in the thermal signature, remotely sensed, in the images. In addition, an opposite change should have occurred in areas of new settlements that originated after the event.

We have found that the Land Surface Temperature (LST) estimation, when rescaled in order to enhance spatial gradients, effectively tracks changes to the urban micro-climate, and can be used to describe the evolution of urban land use after the catastrophic earthquake event was taken into consideration, even when the quality of the image is sub-optimal due to failure of the Scan Line corrector (SCL-off).

Although an assessment of the quantitative effects of the aftermaths was not the main focus of this work (the procedure here uses a very parsimonious model, in terms of input data, in order to precisely estimate land surface temperature), the procedure presented here can be used to perform a qualitative, but correct, description of the dynamics of people’s relocation. 

With this work, we do not intend to propose a novel method for LST estimation, but, rather, we set up a pragmatic, robust, and easy-to-use work-flow, which can be replicated by non-technical users, ensuring the conceptual correctness and reproducibility of image processing. One important point to underline here is that the ability to apply this work-flow to Landsat scenes is limited to the availability of the Atmospheric Correcting Parameters of thermal bands (average atmospheric transmission, effective bandpass upwelling radiance, and effective bandpass down-welling radiance), which can be easily obtained using free web services, or can be directly implemented into the workflow using the Application Programming Interface (API) available with the MODTRAN6 model, with enhanced physics features, including a line-by-line (LBL) algorithm [33,34,35,36].

Clearly, the overall precision and performance of the work-flow could be improved by using alternative and more sophisticated algorithms for the estimation of LST; however, this step can only be performed with a procedure of validation of the output maps with near-sensed measures, when available. These data were not available to us at the time of writing, and this step is envisaged for inclusion in future work.

The authors are aware that it is impossible to attribute all surface temperature variability of an affected or unaffected landscape to earthquake effects. Certainly, there are effects of differential solar zenith angles, and other ambient weather variables, due to the non-identical temporal scale of images on potential surface temperature variability. For instance, the results presented in Figure 9 show over a month difference between March 2009, and May 2010, which may have accounted for some of the spatial variations in the surface temperature of the study area. However, as noted in the Abstract, the authors managed to use ¥ Landsat archive images to provide relative thermal change indications in the study area, which is useful to understand the relative urban changes (more than in finding absolute values of temperatures). Additionally, as stated, all images were acquired at almost the same time (around 8:37 a.m., local time, GMT + 1) when heating was surely being used in houses and offices, for all the four images, and sunshine had just started to warm the ground. If this work captures the interest of both local and national governance, the procedure presented here could be encapsulated in a general-purpose GIS software package as a Quantum-GIS (QGIS) plug-in, an R package, or a GRASS module, in order to provide an additional tool, such as a black-box, for decision-makers. 

It is worth mentioning that this black-box, if the GNU (General Public License) is granted with the software, could be improved in a “community driven way”; the more this software is used, the more weak points of the work-flow can be tackled, and new approaches to LST estimation can be integrated into the software, as well as alternative algorithms for the atmospheric correction of Landsat scenes and for cloud cover assessment. 

Our proposed work-flow could be used retrospectively, as in this paper, or online, after the occurrence of similar disastrous events. In this case a specialization of the algorithm for the Landsat 8 product should also be considered. The usage of a pool of free and open source software and freely-available data sources greatly lowers the total cost for the adoption of the procedure presented in this paper, and could help in “search and rescue” (SAR) campaigns, especially in third-world countries or in remote regions that are difficult to reach.

Finally, the use of demographic and economic data, when available, could provide an additional source of information for a more precise description of urban micro-climate evolution after a disastrous event. 

## Figures and Tables

**Table 1:** Descriptive attributes of the Landsat Enhanced Thematic Mapper Plus (ETM+) images.

Id	Date	Greenwich Medium Time (GMT)	Lat (Deg.)	Lon (Deg.)	LST Successfully Processed
LE71900312008084ASN00	24/03/2008	09:37:21	41.76665	13.61165	
LE71900312008132ASN00	11/05/2008	09:37:13	41.76504	13.59003	
LE71900312008308ASN00	03/11/2008	09:36:11	41.76288	13.63519	
LE71900312008324ASN00	19/11/2008	09:36:25	41.76024	13.64066	
LE71900312009086ASN00	27/03/2009	09:37:26	41.76393	13.6099	*
LE71900312009134ASN00	14/05/2009	09:37:38	41.76234	13.59009	*
LE71900312009278ASN00	05/10/2009	09:37:18	41.76517	13.60086	*
LE71900312009326ASN00	22/11/2009	09:37:54	41.76312	13.65504	
LE71900312010089ASN00	30/03/2010	09:39:24	41.75448	13.58893	
LE71900312010121ASN00	01/05/2010	09:39:23	41.75542	13.62054	*
LE71900312010249ASN00	06/09/2010	09:39:29	41.75601	13.62678	

* Successfully processed.
